# Advanced Heart Failure Therapies in Carceral Settings: LVAD Evaluation in an Incarcerated Patient

**DOI:** 10.1016/j.jaccas.2025.105784

**Published:** 2025-10-18

**Authors:** Sean Halloran, William Weber, Jane Wilcox, Duc Pham, Esther Vorovich, Erin Martin, Rina Murao, Sarah J. Russe, Sarah Chuzi

**Affiliations:** a Department of Medicine, Northwestern University Feinberg School of Medicine, Chicago, Illinois, USA; b Department of Emergency Medicine, Rush University, Chicago, Illinois, USA; c Division of Cardiology, Department of Medicine, Northwestern University Feinberg School of Medicine, Chicago, Illinois, USA; d Division of Cardiac Surgery, Department of Surgery, Northwestern University Feinberg School of Medicine, Chicago, Illinois, USA; e Medical Ethics Program, Northwestern Memorial Hospital, Chicago, Illinois, USA

**Keywords:** cardiac assist devices, chronic heart failure, left ventricle, systolic heart failure

## Abstract

**Clinical Condition:**

Evaluation for advanced heart failure (HF) therapy, including left ventricular assist device (LVAD) placement, in an incarcerated patient.

**Key Questions:**

The key questions are the following: 1) how does incarceration impact cardiovascular risk?; 2) how does incarceration affect advanced HF therapy evaluation, and what legal and ethical considerations apply?; 3) what are the logistical challenges of managing LVAD therapy in the carceral setting?; and 4) what legal pathways exist for medically complex incarcerated patients, and how can clinicians advocate effectively on their behalf?

**Outcome:**

The patient was approved for LVAD placement. Shortly before LVAD implantation, he was approved for medical parole. He successfully underwent LVAD placement, was discharged home, and is now undergoing evaluation for heart transplantation.

**Take-Home Messages:**

Incarceration should not exclude patients from advanced HF therapies. Medical parole should be considered when ongoing care exceeds a facility's capabilities.

## Visual Summary

**Figure:**
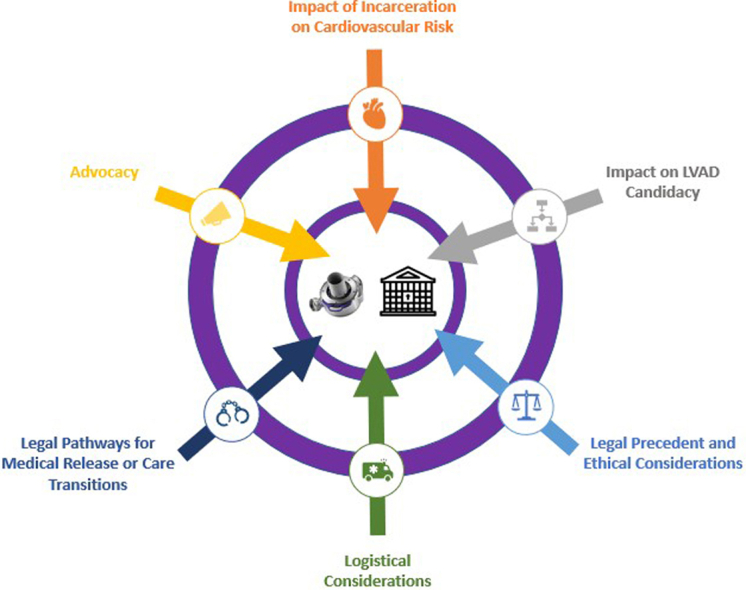

A 36-year-old man with a history of heart failure (HF) secondary to dilated cardiomyopathy presented to our hospital with persistent HF symptoms despite optimization of medical therapy. Six months earlier, he had presented to another hospital in cardiogenic shock requiring inotropic support and was found to have a left ventricular ejection fraction of 10%. Work-up revealed a severely dilated left ventricle (7.0 cm), cardiac index of 1.53 L/min/m^2^, and no evidence of coronary artery disease. He was 18 years into a prison sentence with approximately 50 years remaining. Attempts to transfer the patient to a facility offering advanced HF surgical therapies were unsuccessful at that time due to his incarceration status. As a result, inotropic therapy was weaned, and he was initiated on low-dose guideline-directed medical therapy, including spironolactone 12.5 mg daily and losartan 12.5 mg daily, and a diuretic. The patient was subsequently followed in an outpatient cardiology clinic, with transportation provided by the prison facility. He was transitioned to sacubitril-valsartan 24/26 mg twice daily, and empagliflozin 10 mg daily was added to his regimen.

**Take-Home Messages**

• Incarceration should not exclude patients from advanced HF therapies.
• Medical parole should be considered when ongoing care exceeds a facility's capabilities.

The patient was referred to our hospital by the prison medical team due to worsening HF symptoms. On presentation, he was hemodynamically stable but exhibited signs of volume overload. An echocardiogram was unchanged from prior. Given his progressive symptoms and lack of response to maximally tolerated guideline-directed medical therapy, our multidisciplinary team initiated an advanced HF therapy evaluation. We subsequently present key questions that arose during this evaluation and our approach to addressing each.

## Question 1: How Does Incarceration Impact Cardiovascular Risk?

Incarceration is a significant social determinant of cardiovascular disease (CVD), with intersecting factors such as race, prolonged confinement, and limited access to preventive care, amplifying risk. Studies have demonstrated that incarcerated individuals have higher rates of cardiovascular (CV) risk factors, including hypertension, diabetes, and smoking, compared with the public. These risk factors contribute to significantly worse CV outcomes. In the National Longitudinal Survey of Youth, history of incarceration was associated with more than twice the odds of developing heart disease.^1^

Certain factors, such as race and length of incarceration, further compound these risks. Black men experience higher CV mortality than non-Black men, a disparity that persists even after adjusting for socioeconomic and CVD risk factors.^2^ Long-term incarceration is associated with accelerated biological aging, whereby each year spent in prison may reduce life expectancy by up to 2 years.^3^ Our patient's identity as a Black man experiencing prolonged confinement placed him at the intersection of two powerful and overlapping health disparities. At the same time, the US carceral population is aging rapidly: between 2008 and 2022, the proportion of incarcerated individuals ≥55 years of age increased by 67%, with projections indicating that by 2030, one-third will be ≥55 years of age.^4^ As the US prison population ages, the burden of CVD within the carceral setting will increase substantially. These projections highlight the urgent need for targeted CVD prevention, screening, and treatment in carceral settings.

## Question 2: How Does Incarceration Affect Advanced HF Therapy Evaluation, and What Legal and Ethical Considerations Apply?

Standard evaluation for advanced HF therapies, including heart transplantation and left ventricular assist devices (LVADs), typically considers psychosocial factors such as medication adherence, follow-up capability, social support, and housing stability. Although current guidelines recommend a comprehensive psychosocial evaluation, they do not explicitly address incarceration.^5^ For this patient, incarceration posed a unique challenge to evaluation and eligibility.

Well-established legal and ethical precedent supports access to medical care for incarcerated individuals. In *Estelle v Gamble* (1976),^6^ the US Supreme Court ruled that “deliberate indifference” to serious medical needs violates the constitution, affirming incarcerated individuals' constitutional right to care. Internationally, the Nelson Mandela Rules (2015)^7^ state that individuals deprived of liberty must receive health care that is “equivalent” to that available in the community. Together, these standards demonstrate that incarceration status should not preclude access to advanced HF therapies or other medically necessary treatments. Our multidisciplinary team considered the ethical principle of Justice, which requires that individuals receive equitable treatment, and that one person is not advantaged or disadvantaged based on nonclinical factors. Members of our team outlined our approach and ethical framework for this case in the Hasting Center Report article “Should an Incarcerated Patient get an Advanced Heart Therapy?”^8^ In the current report, we build on that framework by providing a more in-depth analysis of the patient's medical situation and the logistical steps required to implement the care in practice.

The decision between heart transplantation and LVAD therapy required consideration of both clinical and contextual factors unique to this patient. In many ways, heart transplantation would have been a superior long-term therapy, as the complexity of LVAD management—including the need for specialized equipment, reliable power sources, wound care, and trained personnel—were anticipated to pose logistical challenges within the correctional setting. Additional concerns, such as environmental safety risks (eg, the potential for altercations within the facility) further complicated the feasibility of LVAD care. However, due to the more stringent eligibility requirements for transplantation, such as the ability to reliably access immunosuppression, and the scarcity of donor organs, our team determined that LVAD therapy offered the most viable and timely path to advanced HF surgical therapy.

## Question 3: What Are the Logistical Challenges of Managing LVAD Therapy in the Carceral Setting?

**Figure 1:**
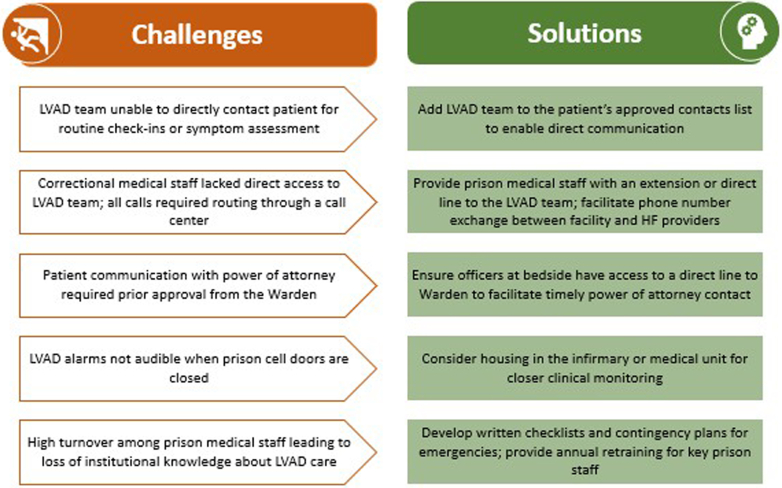
LVAD Care in Carceral Settings: Challenges and Potential Solutions The prison environment can present several logistical and medical challenges to safe LVAD care. Potential challenges should be identified and contingency planning should occur before LVAD placement. LVAD = left ventricular assist device.

In preparation for LVAD implantation, our multidisciplinary team worked with the correctional facility to address the unique logistical challenges of postimplantation care (Figure 1) and develop solutions. This included coordinating training for prison medical staff in LVAD management, ensuring access to necessary equipment (eg, backup batteries, power sources), establishing emergency protocols, and creating a pathway for routine follow-up with the advanced HF team.

The upkeep required for LVADs can strain the supplies and staffing of carceral environments. LVADs require uninterrupted power via wall adapters or batteries and backup batteries available at all times. Carceral facilities often limit what medical equipment may be designated as “keep on person”; for example, extra batteries may be viewed as a fire hazard or security risk. Without access to a backup power source in their cell, a patient could face life-threatening cardiovascular compromise during a power failure while attempting to seek assistance.

The prison facility determined that this patient could not be housed in the general prison population due to concerns that LVAD components (specifically batteries and driveline cords) could be used as weapons. As a result, housing options were limited to medical housing or protective custody, both of which are socially isolating and restrict access to visitation, work, fitness, and educational opportunities. Consequently, a patient's daily life in prison may change significantly after LVAD implantation. These lifestyle and housing implications should be discussed as part of the shared decision-making process.

Incarceration also raises concerns about infection control. LVADs carry an inherent risk for infection, particularly at the driveline exit site. Carceral environments have been shown to harbor elevated rates of resistant organisms such as methicillin-resistant *Staphylococcus aureus*. For example, the prevalence of methicillin-resistant *S aureus* in New York State prisons has been reported to be up to 10 times higher than in the general population.^9^ In addition, patients are dependent on the carceral health system for dressing supplies. One author (W.W.) is aware of cases in which patients were not provided sufficient dressing materials and resorted to using toilet paper in places of sterile gauze, resulting in skin infections.

With these considerations in mind, we recommend several preparatory steps involving the medical team, patient, and correctional facility before LVAD implantation (Table 1), with a focus on contingency planning and training. Together, the team should review the patient's anticipated daily routine postimplantation to proactively identify risks—particularly related to infection control and physical safety within the prison environment. The prison infirmary should receive a supply list, along with protocols for laboratory monitoring and dressing changes. This step is especially important given the high turnover of medical staff in carceral settings; those initially trained may not be the ones providing long-term care.

**Table 1:** Pre-LVAD Planning in Carceral Settings: Roles and Preparedness Checklist

Stakeholder	Key Actions/Considerations
Medical team	✓ Convene interdisciplinary meeting to discuss clinical, logistical, and ethical concerns ✓ Confirm the patient has a surrogate or POA ✓ Coordinate with prison medical leadership regarding perioperative planning
Patient	✓ Informed of LVAD plan and has seen the equipment ✓ Understands what the surgery entails and postoperative expectations ✓ Understands that they may not be informed of surgery/transport date due to facility security policies
Prison facility	✓ Facilitate on-site LVAD training for medical staff ✓ Review emergency protocols, CPR procedures, and EMS activation plans ✓ Establish direct contact mechanisms with the LVAD team and hospital

CPR = cardiopulmonary resuscitation; EMS = Emergency Medical Services; LVAD = left ventricular assist device; POA = power of attorney.

Additionally, the patient, LVAD team, and facility should collaboratively plan for potential complications (including device malfunction, infection, or safety threats) and outline the appropriate response for each scenario. Local hospitals should be contacted in advance to ensure readiness for managing LVAD-related emergencies, and training should be offered if needed. Finally, the patient should be encouraged to designate a surrogate decision-maker in the event they become unable to make medical decisions for themselves.

## Question 4: What Legal Pathways Exist for Incarcerated Patients With Complex Medical Needs, and How Can Clinicians Advocate Effectively on Their Behalf?

Despite existing medical, ethical, and legal frameworks supporting access to care, certain treatments (eg, LVAD) may be logistically incompatible with the medical capacity of some carceral settings. If the essential requirements for LVAD maintenance and monitoring cannot be met, several advocacy pathways may be considered.

One avenue is transfer to a Federal Medical Center (FMC). FMCs are operated by the Bureau of Prisons and provide long-term medical and psychiatric care to federal inmates with complex conditions. This pathway is limited by eligibility and resource constraints. FMC beds are assigned based on medical acuity; therefore, patients stable on an LVAD may have lower transfer priority. Additionally, FMCs only serve patients in federal prisons; therefore, those in state custody or without federal charges are ineligible.

If appropriate medical care cannot be provided within the correctional setting and transfer to an FMC or equivalent facility is not feasible, many states offer a medical parole process. Medical parole permits the early release of incarcerated individuals who have serious or terminal medical conditions, or whose complex medical needs exceed the capabilities of the prison facility. Medical parole is one form of compassionate release, an umbrella term that also includes elder parole or release due to family circumstances (eg, caregiver for a dependent family member).

Medical parole laws reflect the reality that continued incarceration of patients with terminal illness or permanent disability is costly and does not further public safety. For instance, patients 50 to 65 years of age receiving medical parole had a recidivism rate just over 2% and those >65 years of age had almost no recidivism.^10^ Furthermore, managing complex patients can create a significant burden on medical staff, especially when many facilities struggle with staffing vacancies.

Medical parole lies at the intersection of medicine and law, and physician involvement plays a crucial role. Programs exist where volunteer physicians review the medical records of incarcerated individuals and provide expert testimony for medical or geriatric parole eligibility. Such evaluations bridge knowledge gaps for decision-makers like parole boards, who may be unfamiliar with the complexities of serious medical conditions.

**Figure 2:**
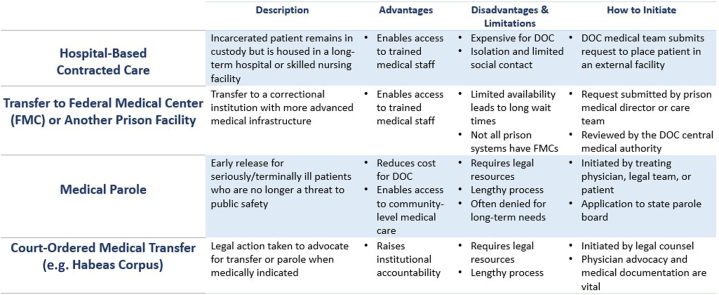
Advocacy Options for Incarcerated Patients With Complex Medical Needs There are several strategies to advocate for incarcerated patients if complex medical needs are not met in prison. Here we present the most common strategies, their strengths and weaknesses, and how to initiate each process. DOC = Department of Corrections; FMC = Federal Medical Center.

If medical parole is denied and significant concerns remain about the adequacy of a patient's medical care in custody, a habeas corpus petition may be pursued. This allows a court to review whether an individual's continued detention is lawful and constitutional (Figure 2). In the context of a patient with an LVAD or other complex medical condition, if the medical or legal team determines that incarceration cannot safely accommodate the necessary care, then continued imprisonment may violate the Eighth Amendment's prohibition against cruel and unusual punishment.

## Outcome

The patient was deemed likely to benefit from LVAD implantation, and our multidisciplinary team collaborated with both the patient and the prison facility to prepare for postimplantation care. However, shortly before the scheduled procedure, the prison facility petitioned the parole board in support of the patient's release, citing concerns about their ability to medically manage a patient with an LVAD. Medical parole was granted, and he subsequently presented to the hospital for surgery after a clear home support plan was conceived. An LVAD was implanted using standard operative technique without intraoperative complications. Vasopressors and inotropes were discontinued on postoperative day 1 and 8, respectively. The remainder of his inpatient course was uncomplicated, and he made a safe transition home. Other than a driveline infection which resolved with treatment, he has not experienced significant LVAD adverse events and is currently undergoing evaluation for heart transplantation.

**Visual Summary:**
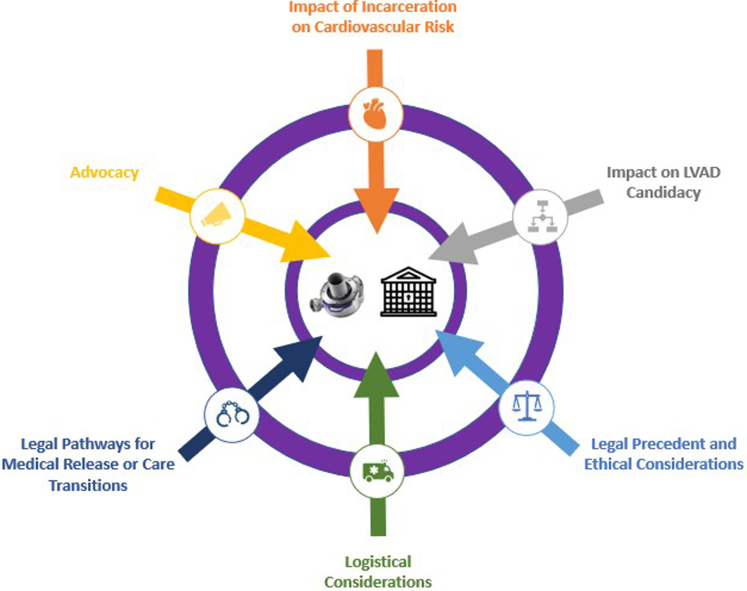
Considerations for LVAD Evaluation and Implantation in Correctional Settings LVAD = left ventricular assist device.

## Funding Support and Author Disclosures

Dr Pham serves as a consultant for AbioMed, Abbott, and Medtronic. Dr Vorovich serves as a consultant for Abiomed, Abbott, and Novo Nordisk. Dr Wilcox serves as a consultant for Abiomed, AstraZeneca, Abbott, Boerhinger Ingelheim, and IONIS. All other authors have reported that they have no relationships relevant to the contents of this paper to disclose.
